# Exogenous sex steroids regulate genital epithelial barrier function in female rhesus macaques

**DOI:** 10.1093/biolre/ioaa105

**Published:** 2020-06-15

**Authors:** Nirk E Quispe Calla, Rodolfo D Vicetti Miguel, Linda Fritts, Christopher J Miller, Kristen M Aceves, Thomas L Cherpes

**Affiliations:** 1 Department of Comparative Medicine, Stanford University School of Medicine, Stanford, CA, USA; 2 California National Primate Research Center, University of California, Davis, CA, USA; 3 Center for Comparative Medicine, University of California, Davis, CA, USA

**Keywords:** estrogen, genital epithelial barrier function, nonhuman primate, progestin

## Abstract

There is concern that using depot-medroxyprogesterone acetate (DMPA) for pregnancy prevention heightens HIV susceptibility. While no clinical data establishes causal link between HIV acquisition and use of this injectable progestin, prior work from our laboratory showed that DMPA comparably lowers genital levels of the cell-cell adhesion molecule desmoglein-1 (DSG1) and weakens genital epithelial barrier function in female mice and women. We likewise saw DMPA increase mouse susceptibility to multiple genital pathogens including HIV. Herein, we sought to confirm and extend these findings by comparing genital epithelial barrier function in untreated rhesus macaques (RM) vs. RM treated with DMPA or DMPA and estrogen (E). Compared to controls, genital tissue from RM with pharmacologically relevant serum levels of medroxyprogesterone acetate displayed significantly lower DSG1 levels and greater permeability to low molecular mass molecules. Conversely, DMPA-mediated effects on genital epithelial integrity and function were obviated in RM administered DMPA and E. These data corroborate the diminished genital epithelial barrier function observed in women initiating DMPA and identify RM as a useful preclinical model for defining effects of exogenous sex steroids on genital pathogen susceptibility. As treatment with E averted DMPA-mediated loss of genital epithelial barrier function, our results also imply that contraceptives releasing progestin and E may be less likely to promote transmission of HIV and other sexually transmitted pathogens than progestin-only compounds.

## Introduction

High rates of maternal and infant mortality and the need for affordable contraception are characteristics common to many parts of the world with large burden of HIV disease, and these conditions combine to make control of the HIV pandemic and pregnancy prevention highly interconnected public health concerns [1]. Entwining the connections further still are clinical reports that indicate women using the injectable progestin depot-medroxyprogesterone acetate (DMPA) are significantly more likely to acquire HIV than women using no hormonal contraception [2, 3]. In particular, DMPA is a popular contraceptive choice in sub-Saharan Africa (SSA), the region of the world where most new HIV infections occur [4, 5]. In SSA, women 15–24 years of age are especially vulnerable, and represent about 25% of the incident infections annually [6].

While adolescent and young women in SSA are disproportionally affected by HIV, the reasons for this are not well defined. However, various explanations have been offered, including gender-based social, economic, political, and cultural disparities, and the high regional prevalence of intergenerational sexual relationships [7–9]. In addition to these possibilities, meta-analytic review of all sufficiently high-quality data from studies exploring the relationship between HIV and hormonal contraception indicated that reproductive age-women using DMPA are about 40% more likely to acquire HIV than women using no hormonal contraceptive [4, 10]. The accumulated weight of this clinical data is persuasive, but it is also possible that increased frequencies of unprotected sexual intercourse among women using hormonal contraception vs. no form of contraception spuriously identified DMPA as a critical HIV risk factor [11].

As rationale for the disproportionate impact of HIV on adolescent and young women in SSA remains ambiguous, additional clinical studies that minimize the impact of confounding behavioral variables on data interpretation are needed. It is also necessary to precisely define biological mechanisms with the potential to alter HIV transmission efficiency in young reproductive-age women. Our laboratory showed that treating mice with DMPA reduced genital levels of desmoglein-1 (DSG1) and other cell-cell adhesion molecules and increased penetration of intravaginally administered low molecular mass molecules and activated leukocytes into genital submucosal tissue [12–14]. We also demonstrated this DMPA-mediated weakening of genital epithelial barrier function respectively increased susceptibility of wildtype and humanized mice to genital infection with herpes simplex virus type 2 (HSV-2) and cell-associated human immunodeficiency virus type 1 (HIV-1) [12–14]. Conversely, DMPA-mediated effects on genital epithelial barrier function and pathogen susceptibility were obviated in mice administered DMPA and exogenous estrogen [12, 13]. Notably, analogous changes in genital epithelial integrity and permeability were identified in DMPA-treated mice and women initiating use of DMPA [12], comparative findings that established mice appropriately model important elements of the human response to this exogenous progestin [12]. To confirm and extend these results to another preclinical model, herein we explored genital epithelial integrity and barrier function in female rhesus macaques (RM) administered DMPA alone or in combination with estrogen-containing compounds.

## Material and methods

### Procedures

**Figure 1:**
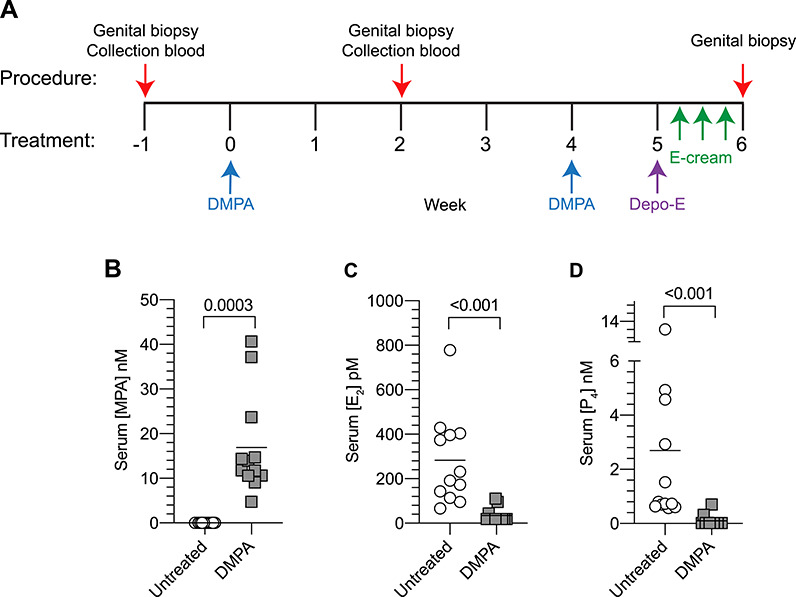
DMPA-treated RM displayed pharmacologically relevant serum MPA concentrations and disruption of the HPO axis. (A) Genital biopsies and peripheral blood samples were obtained from RM 1 week before and 2 weeks after intramuscular injection of 20 mg of DMPA. As indicated, RM received a second DMPA injection and systemic Depo-E or intravaginal E-containing cream before additional biopsies were collected. (B–D) Panels compare serum levels of (B) MPA, (C) E_2_, or (D) P_4_ in untreated and DMPA-treated RM (2 weeks after DMPA injection). Column scatter plots depict results from a single experiment (n = 12 per condition); bars designate means. Statistical analyses were performed using the 2-tailed paired Student *t*-test. DMPA, depot medroxyprogesterone acetate; Depo-E, depot estradiol cypionate; E_2_, estradiol; E-cream, conjugated estrogen-containing vaginal cream; MPA, medroxyprogesterone acetate; P_4_, progesterone; RM, rhesus macaques.

Female RM (*Macaca mulatta*) used in these studies were 5–8 years old and were housed at the California National Primate Research Center. All studies were approved by the University of California–Davis Institutional Animal Use and Care Committee prior to initiation and each procedure performed in accordance with regulations established by the American Association for Accreditation of Laboratory Animal Care. For sedation, RM were intramuscularly (IM) administered 10 mg/kg of ketamine hydrochloride (Parke-Davis, Morris Plains, NJ) or 0.7 mg/kg of tiletamine hydrochloride and zolazepam (Fort Dodge Animal Health, Fort Dodge, IA). After adequate sedation, 10 mL of peripheral blood was collected at indicated time points to quantify serum levels of progesterone (P_4_), estradiol (E_2_), and medroxyprogesterone acetate (MPA) (Figure 1A). Two vaginal biopsies were collected at timepoints indicated in this figure and placed in cold RPMI-1640 (Mediatech, Manassas, VA) supplemented with 10% charcoal/dextran-treated fetal bovine serum (R&D systems, Minneapolis, MN) (hereafter termed transport medium) for use in permeability assays. Two ectocervical biopsies were also obtained, one was immediately immersed in in *RNAlater*™ (Qiagen, Hilden, Germany) and stored at −80°C for use in gene expression studies. The second ectocervical biopsy was placed in 4% buffered methanol-free formaldehyde (Thermo Fisher Scientific, Rockford, IL) and used in histological and immunohistochemical studies.

### Sex steroid treatments

As indicated in Figure 1A, RM were IM administered 20 mg of DMPA (Pfizer, New York, NY). For some studies, an identical 20 mg dose of DMPA was administered 30 days after the first. Also as indicated, RM were IM administered 20 mg of DMPA and 2.5 mg of estradiol cypionate (Pfizer) (hereafter termed Depo-E) or IM administered 20 mg of DMPA and intravaginally administered 1 g of Premarin® (Pfizer), a vaginal cream that contains conjugated estrogens (hereafter termed E-cream). The cream was applied every other day for three total treatments (with the last treatment administered 3 days prior to collection of genital biopsy specimens) (Figure 1A).

### Sex steroid quantification

The Endocrine Technologies Support Core at the Oregon National Primate Research Center quantified serum sex steroid concentrations. Using a Cobas® e 411 Analyzer (Roche Diagnostics, Indianapolis IN), P_4_ and E_2_ levels were measured by electrochemiluminescence [15, 16]. MPA levels were measured by liquid chromatography–tandem triple quadrupole mass spectrometry (LC–MS/MS) as previously described [17].

### Permeability assays

Evaluation of the barrier function of vaginal mucosal epithelium was performed as previously described [12, 13, 18, 19]. In summary, fresh vaginal biopsies were placed in chilled transport medium and transferred to sterile 96-well plates. Tissue was submerged in 50 μL of phosphate buffered saline that contained 62.5 μg of dextran labeled with Texas Red (DR) (MW = 70,000 Da), and 50 μg of Lucifer yellow CH, lithium salt (LY) (MW = 457.2 Da) (Life Technologies, Carlsbad, CA) and incubated 45 min at 37°C/5% CO_2_. Specimens were fixed 24 h in buffered 4% methanol-free formaldehyde and embedded in 6% agarose. Using a PELCO EasiSlicer™ (Ted Pella Inc., Redding, California), 200–300 μm tissue sections were counterstained with DAPI (4′,6-diamidino-2-phenylindole, Life Technologies). Sections were viewed with a Nikon A1 confocal microscope and vaginal mucosal epithelial permeability calculated by fluorescent LY molecule infiltration of the vaginal epithelium (pixels/μm^2^) using ImageJ software [20, 21].

### Histology

Ectocervical tissue was fixed for 48 h in 4% formaldehyde before paraffin embedding. Five μm sections were stained with hematoxylin and eosin (H&E) and imaged with the NanoZoomer 2.0-RS (Hamamatsu Photonics K.K., Hamamatsu City, Shizuoka, Japan). Thickness of the *stratum corneum* in ectocervical epithelium was quantified using NDP.view2 software (Hamamatsu Photonics K.K.). For DSG1 immunohistochemistry, unstained sections were de-paraffinized in xylene and sequentially rehydrated with 100% ethanol, 96% ethanol, and deionized water. Antigen retrieval was performed in citrate buffer with 0.02% Tween 20 (pH = 6.0) for 20 min at 95°C. Endogenous peroxidase activity was quenched in 3% hydrogen peroxidase and sections placed for 4 h with 5% normal goat serum at 4°C (Cell Signaling Technology®, Danvers, MA) and 24 h at 4°C in rabbit anti-DSG1 (clone EPR67766(B), Abcam Inc, Cambridge, MA) diluted 1:100 with SignalStain® antibody diluent (Cell Signaling Technology®). Using manufacturer’s instructions, sections were washed thrice and incubated with SignalStain® Boost detection and SignalStain® DAB Chromogen (both from Cell Signaling Technology®). Sections were counterstained with hematoxylin and placed in mounting medium (Cell Signaling Technology®). For DSG1 protein quantification, images were captured using the NanoZoomer 2.0-RS slide scanner and analyses performed using ImageJ software [20, 21].

### Reverse transcriptase-quantitative PCR (rt-qPCR)

RNA was isolated from ectocervical tissue using the RNeasy Lipid Tissue Kit and RNase-free DNase Set (Qiagen) using manufacturer’s instructions. Samples were re-suspended in nuclease-free water to quantify RNA via spectrophotometer (Molecular Devices, San Jose, CA). 100 ng of RNA were used to generate cDNA using the SuperScript™ IV VILO™ Master Mix (Invitrogen, Thermo Fisher Scientific). For reactions, TaqMan™ Fast Universal PCR Master Mix (Applied Biosystems®) was used in combination with cDNA template and specified primers. Gene expression levels for desmoglein-1 (*DSG1*, Rh02840468_m1), desmocollin 1 (*DSC1*, Rh02856129_m1), occludin (*OCLN*, Rh02842594_m1), tight junction protein 1 (*TJP1*, Rh01551861_m1), claudin 1 (*CLDN1*, Rh01076358_m1) and the housekeeping gene ribosomal protein L32 (*Rpl32*, Rh028117722_s1) [22] (all from Life Technologies, Pleasanton, CA) were quantified by rt-qPCR using the QuantStudio 3 Real-Time PCR Systems (Applied Biosystems®) and the ∆CT method.

### Statistical considerations

Prism 8 software (GraphPad, La Jolla, CA) was used for statistical analyses. Where indicated, normal distribution was tested by D'Agostino & Pearson omnibus test or evaluation of the residuals (when experimental sample numbers were < 8). The paired Student *t*-test was used when comparing two paired groups of samples. For multiple group comparisons, 1-way ANOVA and Dunnett’s multiple comparisons post hoc test was used. For statistical comparisons, *P*-values ≤ 0.05 were deemed statistically significant.

## Results

### DMPA-treated RM displayed pharmacologically relevant serum MPA concentrations at the time genital biopsies were collected

Prior publications identified serum MPA levels of 21—25 nM in pigtail macaques (*Macaca nemestrina*) that 2–3 weeks earlier had been IM administered 15–20 mg of DMPA [23, 24]. To quantify serum concentrations of MPA in the current study, peripheral blood was collected 1 week before and 2 weeks after RM were IM administered 20 mg of DMPA. In the DMPA-treated RM, a mean serum MPA value of 16.9 nM (range 4.7–40.6 nM) was identified (Figure 1B). As peak serum MPA levels of 5.8–62 nM are detected in women IM administered 150 mg of DMPA [25], current results established that pharmacologically relevant serum MPA levels were circulating at the time genital biopsy specimens used to evaluate gene expression and epithelial barrier function were obtained.

### DMPA significantly reduced serum levels of endogenous E_2_ and P_4_

Clinical research data indicates that DMPA provides contraception primarily by interrupting the hypothalamic-pituitary-ovarian (HPO) axis [26]. Prior pharmacokinetic studies further identified that during a single 3-month interval after DMPA injection, women display mean serum E_2_ concentration around 18.9 pg/mL [27]. Using blood collected 1 week before and 2 weeks after RM were IM administered 20 mg of DMPA, we respectively detected mean serum E_2_ levels of 77 (282 pM) and 9.4 pg/mL (34.5 pM) before and after DMPA treatment (Figure 1C). In DMPA-treated animals, we also detected a mean serum P_4_ levels of 847 pg/mL (2694 pM) before treatment and 70 pg/mL (98 pM) after treatment (Figure 1D). Together, these findings offered evidence for HPO axis disruption in animals IM administered 20 mg of DMPA 2 weeks previously.

### DMPA significantly reduced genital levels of the cell-cell adhesion molecule DSG1

**Figure 2:**
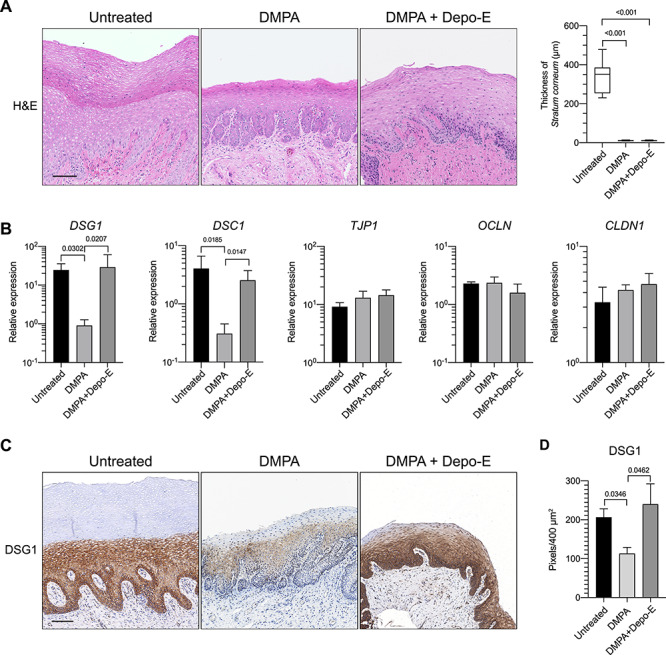
Exogenous sex steroids regulate genital desmosomal cadherin expression. Ectocervical biopsies were obtained from RM prior to treatment, after DMPA administration, or after DMPA and Depo-E administration as depicted in Fig. 1A. (A) Representative images from H&E-stained sections used to measure *stratum corneum* thickness (200× magnification) are displayed on left. Panel on right shows *stratum corneum* thickness significantly decreased in ectocervical specimens obtained after treatment with DMPA or DMPA and Depo-E. (B) rt-qPCR assays show ectocervical *DSG1* and *DSC1* expression significantly reduced by DMPA treatment vs. no treatment or treatment with DMPA and Depo-E, whereas DMPA treatment did not significantly affect gene expression of the cell-cell adhesion molecules *OCLN*, *TJP1,* and CLDN1. (C) Representative images from immunohistochemistry staining used to define DSG1 protein expression (magnification 200×). (D) Congruent with *DSG1* gene expression, DSG1 protein levels were significantly increased in biopsies collected pretreatment or after DMPA and Depo-E treatment vs. samples obtained after DMPA treatment alone. Box and whisker plots in (A) show median value ± range. Bars in (B) and (D) bar graphs denote mean ± SD. Statistical analyses performed using 1-way ANOVA and Dunnett’s multiple comparisons post hoc test. DMPA, depot medroxyprogesterone acetate; DSG1; desmoglein 1, DSC1; desmocollin 1, *OCLN;* occludin, *TJP1*; tight junction protein 1, *CLDN1;* claudin 1, Depo-E, depot estradiol cypionate. Image scale bars denote 100 μm.

As all DMPA-treated RM in the current study displayed HPO axis interruption with pharmacologically relevant serum MPA levels, genital biopsies obtained from untreated and DMPA-treated animals were used to define the effects of progestin treatment on genital epithelial integrity. We also examined this response in tissue collected from RM administered DMPA and an estrogen (E)-containing compound. In analyses of H&E-stained ectocervical sections, *stratum corneum* thickness was significantly reduced in RM administered DMPA or DMPA and Depo-E compared to untreated controls (Figure 2A). Quantifying ectocervical gene expression in these same three groups, we found significantly lower *DSG1* and *DSC1* levels in RM treated with DMPA alone, whereas treatment with DMPA and Depo-E restored *DSG1* and *DSC1* expression to levels seen in untreated animals (Figure 2B). As opposed to the effects of DMPA and Depo-E on *DSG1* expression, there were no statistically significant between-group differences in ectocervical expression of multiple other cell-cell adhesion molecules, including *TJP1*, *CLDN1*, and *OCLN* (Figure 2B). Congruent with rt-qPCR findings for *DSG1* expression, however, we saw significantly higher levels of DSG1 protein in ectocervical tissue from untreated controls and RM treated with DMPA and Depo-E vs. RM injected with DMPA alone (Figure 2C and D). When considered with earlier reports from our laboratory, these findings established that DMPA treatment induces comparable effects on genital *DSG1*, *DSC1*, *TJP1*, *CLDN1*, and *OCLN* gene expression in mice and RM [12–14]. Moreover, current findings offered novel indication that exactly analogous changes in *DSG1* expression and DSG1 protein levels occur in the ectocervix of DMPA-treated RM and women initiating use of this injectable progestin [12].

### DMPA significantly compromised genital mucosal barrier function

**Figure 3:**
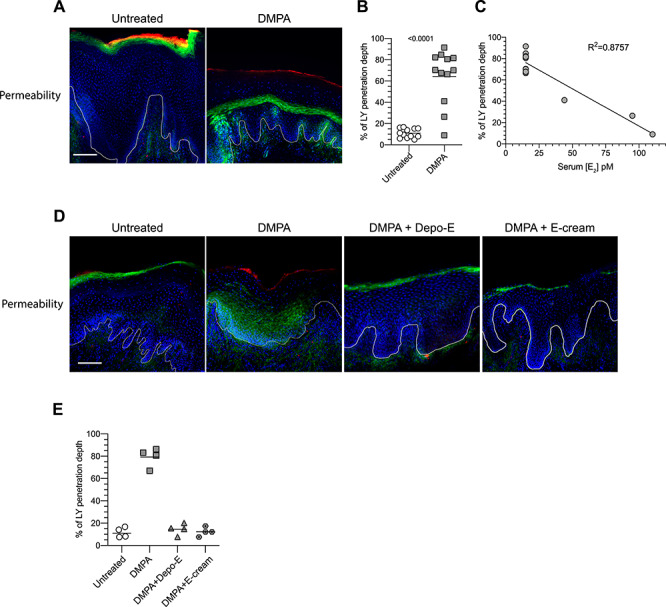
Exogenous sex steroids regulate genital mucosal barrier function. (A) Representative confocal microscopy images depict permeability of vaginal mucosal epithelium to penetration by low molecular mass molecules before and after DMPA treatment. Permeability assay described in Materials and Methods section; lucifer yellow (LY) (green); 70 kDa dextran labeled with Texas Red (red), DAPI (blue). (B) Depth of LY entry into the vaginal mucosal epithelium was significantly increased after DMPA treatment. (C) Linear regression analysis denotes LY infiltration depth into vaginal mucosal tissue of untreated and DMPA-treated RM was inversely related to serum E_2_ levels. (D) Representative confocal microscopy images illustrate greater LY penetration into vaginal mucosa of DMPA-treated RM vs. no treatment, DMPA and Depo-E treatment, or DMPA and E-cream treatment. (E) Panel shows significantly reduced permeability of the vaginal mucosa in RM administered DMPA and systemic or intravaginal E vs. DMPA alone. Column scatter plots depict measurements for single RMs; bars designate means. Statistical analyses were performed in (B) with the 2-tailed paired Student *t*-test and in (E) the 1-way ANOVA and Dunnett’s multiple comparisons post hoc test compared the DMPA treatment group to all other groups (all *P*-values were < 0.0001). DAPI; 4′,6-diamidino-2-phenylindole; DMPA; depot medroxyprogesterone acetate, Depo-E, depot estradiol cypionate; E_2_, estradiol; E-cream, estrogen-containing vaginal cream. Scale bars on images denote 100 μm.

Because treatment of RM with DMPA significantly reduced genital levels of the desmosomal cadherins DSG1 and DSC1, we posited that treatment similarly compromises genital mucosal barrier function. As hypothesized, vaginal biopsies from untreated and DMPA-treated RM showed ex vivo penetration of LMM molecules into the vaginal epithelium was significantly enhanced by DMPA treatment (Figure 3A and B). Moreover, the depth of LMM molecule penetration in these confocal microscopy studies was inversely correlated with serum E_2_ concentrations measured at the time of vaginal biopsy collection (Figure 3C), results that suggested a role for endogenous E in promoting or maintaining genital epithelial barrier function. Based on this possibility, we concluded the current investigation by defining the capacity of exogenous E to restore genital epithelial barrier function in DMPA-treated animals. As predicted by the levels of DSG1 protein in RM administered DMPA vs. DMPA and Depo-E, these studies showed that genital mucosal epithelial barrier function was significantly enhanced in RM administered DMPA and Depo-E or E-cream vs. RM treated with DMPA alone (Figure 3D and E).

## Discussion

Clinical investigation faces considerable obstacles to illuminate a clear path forward regarding the use of DMPA in areas of the world where large numbers of women are living with HIV, an observation that implies alternative research strategies will be needed to help define this relationship. Whereas multiple reports in the past 35 years showed enhanced HIV risk in women using DMPA, all of these data were acquired from observational studies [4, 10]. Representing the first randomized clinical trial to explore relationships between HIV acquisition and this injectable progestin, the Evidence for Contraceptive Options and HIV Outcomes (ECHO) Trial compared HIV risk in women randomized to initiate use of DMPA, levonorgestrel (LNG) implant, or the copper intrauterine system (Cu-IUS) [28]. With results published in 2019, study investigators concluded these contraceptive choices (when compared to one another) are not statistically significant HIV risk factors [29]. However, interpretation of data in the ECHO Trial is made problematic because HIV risk in DMPA users was defined relative to risk in women using LNG implant or Cu-IUS, contraceptives that have unknown effects on HIV susceptibility [30]. Moreover, while prior observational studies indicated HIV risk is increased by about 40% by DMPA use [4, 10], the ECHO Trial had been powered to detect hazard ratios larger than 50% [29]. This study design decision makes it particularly challenging to understand the biological significance of ECHO Trial results that show risk of HIV infection increased about 29% in women using DMPA vs. Cu-IUS [29].

While performing more clinical trials will help define actual relationships between HIV and individual forms of contraception, the ability to appropriately model changes in antivirus immunity produced by these contraceptives can clarify these links and provide findings unobtainable in clinical research. Our current results show that RM model important elements of the human host response to DMPA and establish this progestin significantly reduces genital levels of the desmosomal cadherins DSG1 and DSC1 and impairs genital epithelial barrier function. Moreover, these effects are produced in RM with pharmacologically relevant serum levels of MPA. In support of their clinical relevance, current findings mirror the lower DSG1 levels and compromise of genital epithelial barrier function generated in women initiating DMPA or LNG-IUS use [12, 19, 31]. Conversely, the loss of genital epithelial thickness induced by DMPA was more pronounced compared to that produced in RM by the menstrual cycle fluctuation in endogenous progesterone and estrogen levels [32]. This difference is likely the result of more sustained and lower circulating E_2_ levels induced by DMPA vs. the normal menstrual cycle and a stronger agonistic effect of MPA vs. endogenous progesterone on the progesterone receptor [33]. Unlike genital tissue obtained from RM at any menstrual cycle stage [34], we also saw significant loss of desmosomal cadherin expression and genital epithelial barrier function in DMPA-treated vs. untreated RM. These differences between normally cycling and DMPA-treated RM are consistent with the role played by desmosomes in promoting epithelial resistance to mechanical insult [35] as well as the profound loss of epithelial barrier function in humans and mice with DSG1 deficiency [36–38]. Current and prior reports from our laboratory thus show DMPA comparably downregulates genital desmosomal cadherin expression in mice, RM, and humans [12–14] and highlight the shared evolutionary path for these proteins [39].

Also congruent with our mouse model findings [12], current RM data indicates that there is limited correlation between genital epithelial barrier function and overall genital epithelium thickness or recovery of the *stratum corneum*. Together, these animal model findings challenge the notion that the *stratum corneum* is an all-important component of the genital epithelial barrier [34, 40] and suggest that the progestin-mediated effects on genital pathogen susceptibility are more dependent on an impaired genital epithelial barrier than presence of the *stratum corneum* or overall genital epithelial thickness. While we are currently exploring the latter possibility in RM SIV challenge studies, our current findings already establish that DMPA-mediated changes to genital epithelial integrity and barrier function are reversed by concomitant administration of E-containing compounds. These results are congruent with mouse model data that showed combined treatment with progestin and E avoided the compromise of genital epithelial barrier function and uniform susceptibility to genital HSV-2 or HIV-1 infection produced by DMPA treatment alone [12, 13, 18]. Whereas RM administered DMPA or DMPA and E had comparable *stratum corneum* thickness, exogenous E prevented DMPA-mediated increases in genital epithelial permeability. These findings may result from partial inhibition of E-mediated epithelial proliferation and differentiation induced by strong agonistic effects of MPA on the progesterone receptor [41, 42]. Alternatively, these findings may result from known effects of E on *DSG1* expression, desmosome organization, and maintenance of genital epithelium integrity in mice, RM, and humans [43–46], presumably through stimulation of E receptors in genital epithelial cells [43, 47].

In summary, results from the current investigation identify the RM as an appropriate preclinical model for exploring the effects of sex steroids on genital epithelial barrier function. Our findings also imply that RM studies are well-positioned to illuminate sex steroid-mediated effects on genital pathogen susceptibility and help delineate contraceptive strategies least likely to promote genital infection. As significant hurdles are in place that will continue to impede clinical studies from defining precise relationships between HIV susceptibility and contraception, RM and mouse models seem indispensable for informing both clinical study design and recommendations for contraceptive choice in regions with large HIV burden and for providing women worldwide the type of data needed to make reasoned decisions regarding contraceptive selection.
